# Influence of the Epoxy Resin Process Parameters on the Mechanical Properties of Produced Bidirectional [±45°] Carbon/Epoxy Woven Composites

**DOI:** 10.3390/polym13081273

**Published:** 2021-04-14

**Authors:** Claudia A. Ramírez-Herrera, Isidro Cruz-Cruz, Isaac H. Jiménez-Cedeño, Oscar Martínez-Romero, Alex Elías-Zúñiga

**Affiliations:** 1 Mechanical Engineering and Advanced Materials Department, School of Engineering and Science, Tecnologico de Monterrey, Ave. Eugenio Garza Sada 2501 Sur, Monterrey 64849, Mexico; claudia.ramirez@ciqa.edu.mx (C.A.R.-H.); isidro.cruz@tec.mx (I.C.-C.); oscar.martinez@tec.mx (O.M.-R.); 2 Engineering Department, Safran Mexico, Av. Ishikawa 1001 (Esquina con Av. Taguchi) Parque Industrial Supra, Chihuahua 31183, Mexico; isaac-h.jimenez-c@safrangroup.com

**Keywords:** epoxy resin, curing process, FTIR spectroscopy, woven carbon fiber fabric composites, mechanical performance

## Abstract

This work focuses on investigating the curing process of an epoxy-based resin—Aerotuf 275-34^TM^, designed for aerospace applications. To study the curing degree of Aerotuf 275-34^TM^ under processing conditions, woven carbon fiber fabric (WCFF)/Aerotuf 275-34^TM^ composite laminates were produced by compression molding using different processing temperatures (110, 135, 160, and 200 °C) during 15 and 30 min. Then, the mechanical behavior of the composite laminates was evaluated by tensile tests and correlated to the resin curing degree through Fourier-transform infrared spectroscopy (FTIR) analysis. The results show the occurrence of two independent reactions based on the consumption of epoxide groups and maleimide (MI) double bonds. In terms of epoxide groups, a conversion degree of 0.91 was obtained for the composite cured at 160 °C during 15 min, while the measured tensile properties of [±45°] WCFF/Aerotuf 275-34^TM^ laminates confirmed that these epoxy resin curing processing conditions lead to an enhancement of the composite mechanical properties.

## 1. Introduction

Because of their physical and mechanical properties, thermosetting polymers cured with epoxy resins have found a broad range of applications, mainly as matrices in advanced composites for aircraft and automotive applications [1,2,3,4,5]. Some of major attributes of these materials are as follows: (i) excellent chemical resistance, particularly to alkaline environments; (ii) outstanding adhesion to a broad variety of substrates; (iii) high tensile, compressive, flexural, and fatigue strengths; (iv) low shrinkage; (v) excellent electrical insulation properties; (vi) good corrosion resistance; and (vii) ability to cure over a wide range of temperatures [4,6]. To take advantage of epoxy resins properties, it is important to understand their curing kinetic process to improve composites’ processing time, energy consumption, costs, and product quality [7,8]. Therefore, the curing process of epoxy resin is the most crucial stage of the manufacturing process as it influences the final properties of fabricated parts. Curing or cross-linking processes of epoxy resins take place through the oxirane functional monomers or hydroxyl groups to form a three-dimensional infusible network. The cross-linking process can occur mainly by two types of curing mechanisms: direct coupling of the resin molecules by a catalytic homopolymerization or coupling through an intermediate reactive known as curing agent, which are compounds containing active hydrogen (polyamines, polyacids, polymercaptans, and polyphenols, to name a few) [5,6,7]. Owing to the high reactivity of chemical species involved in the curing process, the reaction is highly exothermic; therefore, it is imperative that the polymerization process of epoxy resins must be carefully controlled in order to prevent that the released reaction heat can self-accelerate the process [2,4]. Several analytic techniques have been demonstrated to monitor the extent of the reaction to determine the curing degree of epoxy resins [2,3,4,7,8,9,10,11,12,13,14]. Among direct techniques, Fourier-transform infrared spectroscopy (FTIR) can be used to evaluate the concentration of one or more reactive groups as a function of time (during the curing process). FTIR is mainly used because of its versatility, practicality, and nondestructive methodology. In addition, this technique offers outstanding advantages, unlike other characterization tools. Furthermore, the FTIR vibrational spectrum is not influenced by the state transitions of the resin, which allows monitoring the whole curing stages, from the fluid mixture to the glassy resin [15,16]. By analyzing the FTIR absorption bands, it is possible to assess all the concentration profiles of the chemical species present in the resin system, obtaining direct information about the reaction mechanisms involved in the curing process [16]. Moreover, FTIR allows to estimate the influence of the parameters associated with the resin curing process such as temperature, time, resin/hardener ratio, and additives, among others, on the conversion degree.

Some previous research works that study the curing process of epoxy resins using FTIR spectroscopy can be found in [2,7,9,15,16,17,18,19]. However, research works that focus on validating the epoxy resins processing parameters and their influence on the mechanical properties of parts fabricated for the aerospace sector are still scarce [3].

Therefore, this article focuses on studying the influence that the curing degree of Aerotuf 275-34TM resin has on woven carbon fiber fabric [±45°] (WCFF)/Aerotuf 275-34TM composite laminates produced by compression molding, using different processing temperatures (110, 135, 160, and 200 °C) during time periods of 15 and 30 min. Furthermore, tensile tests were performed on [±45°] WCFF/Aerotuf 275-34^TM^ composite laminate samples to correlate their mechanical properties with the resin curing degree.

## 2. Materials and Methods

### 2.1. Materials

Aerotuf 275-34^TM^, henceforth simply denoted as Aerotuf, provided by a local company was used in this study as impregnation resin. [±45°] WCFF prepreg produced from ToraycaTM T700GC carbon fiber yarns (Toray Industries, Inc., Tokyo, Japan) and Aerotuf having ~60 wt.% of fiber content was used for the manufacturing of the composite laminates. The epoxy resin and the prepreg were stored at −20 °C. Ease Release 200 (Mann Release Technologies, Inc., Macungie, PA, USA) was used as a mold release agent.

### 2.2. [±45°] WCFF/Aerotuf Composite Laminates

[±45°] WCFF/Aerotuf composite laminates using different processing conditions were prepared. Here, the angle between the carbon fibers and the direction of the applied stress during the mechanical characterization is to referred as [±45°]. First, WCFF prepreg laminates with carbon fibers oriented at [±45°] were stacked into a flat mold of 25 cm length and 13.5 cm width. Then, the mold was placed into an oven at 100 °C for 15 min to reduce the viscosity of the resin. Next, the preheated preform was compressed at a fixed pressure of 8.3 MPa using a Bench Top Laboratory Manual Press, model 4128 (Carver, Inc., Wabash, IN, USA). Prior to FTIR and mechanical characterization, uncured resin was analyzed by DSC at 10 °C min^−1^ in order to select the appropriate working temperature values. From these measurements, the exothermic peak was recorded in the temperature range of 130–180 °C. Thus, the curing temperatures were chosen within this range, and two temperature measurements were collected below and above this temperature range. The curing temperature was set at the values of 110, 135, 160, and 200 °C, and the curing time was fixed at 15 and 30 min. Upon the completion of the curing time, laminates were cooled down, keeping the same molding pressure. All the composite laminates produced at different curing degrees were cut in rectangular specimens using an abrasive water-jet cutter according to the specified dimensions in ASTM standard D3039 (250 × 25 × 2.5 mm^3^) for further mechanical characterizations on a Shimadzu Universal/Tensile Testing Machine (Shimadzu Corporation, Kyoto, Japan). Measurements were performed at room temperature using a crosshead rate of 2 mm min^−1^. Average values of ultimate tensile strength (UTS) and elastic modulus, E, were obtained averaging five specimens’ data for each composite laminate.

### 2.3. Aerotuf Curing Process

To investigate the Aerotuf curing process, FTIR spectroscopy measurements were performed at a spectral range of 4000–500 cm^−1^ and resolution of 4 cm^−1^ in a Frontier FTIR/FIR system (Perkin Elmer, USA) in attenuated total reflectance (ATR) mode using a universal ATR accessory. The evaluation of some cured resin samples taken from the composite laminates and prepared at different processing conditions confirms that the applied pressure does not affect the curing process, which agrees with the results found in [20].

## 3. Results and Discussion

### 3.1. Functional Groups Analysis of Aerotuf

**Figure 1:**
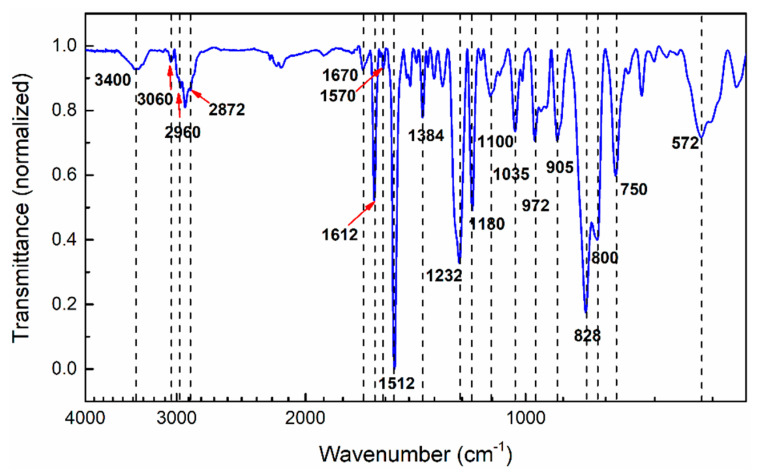
Fourier-transform infrared spectroscopy (FTIR) spectrum of uncured Aerotuf resin. Notice that, at high wavenumber values, the FTIR spectrum shows an absorption band located at 3400 cm^−1^ attributed to O–H stretching mode of hydroxyl groups, revealing the presence of dimers or high molecular weight species.

The chemical nature of Aerotuf has not been previously reported in the literature. Therefore, an analysis of their functional groups by FTIR spectroscopy was performed. Figure 1 shows the normalized FTIR spectrum of uncured Aerotuf and Table 1 summarizes the assignments for their main absorption bands. Notice from Figure 1 that, at high wavenumbers, the FTIR spectrum shows the presence of an absorption band located at 3400 cm^−1^ attributed to O–H stretching mode of hydroxyl groups, revealing the presence of dimers or high molecular weight species. Bands located at 3060 cm^−1^ are assigned to C–H stretching mode of the epoxide group, while those located between 2960 and 2872 cm^−1^ correspond to –CH_2_ and –CH_3_ stretching vibration modes of aromatic and aliphatic chains, respectively. Some bands of weak intensity located at 1670, 1570, and 1384 cm^−1^ evidence the existence of N–H functional groups of amines, and C–N bonds of amine and imide groups possibly belonging to the catalyst and modifier added to the modified epoxy resin [21]. Absorption bands from medium to strong intensity centered at 1612 and 1512 cm^−1^ correspond to C=C and C–C stretching vibrations of benzene rings present in the monomer backbone chain. Furthermore, C–O–C and C–O stretching vibrations attributed to ether linkage are confirmed by the presence of bands at 1232, 1180, and 1035 cm^−1^. The epoxide groups present in the resin are identified from the characteristic absorption bands centered at 972 and 905 cm^−1^, which are associated to the glycidyl ether functionality and the C–O stretching vibration mode of oxirane ring, respectively. At lower wavenumbers, the sharp band between 828 and 800 cm^−1^ is assigned to the out-of-plane bending of the H–C= vibration present in maleimide (MI) units and 1–4 substituted aromatic rings. Finally, bands located between 750 and 572 cm^−1^ were attributed to C–H out-of-plane absorptions of substituted aromatic rings. Some other characteristic absorption bands of amine and imide groups such as N–H symmetric stretching of primary amine at ~3380 cm^−1^ and imide carbonyl stretching mode at 1714 cm^−1^ are not completely resolved in the spectrum, mainly owing to their overlapping with the O–H stretching band or, in the case of MI compounds, owing to their complete dissolution at molecular level in the epoxy/amine system, which prevents the presence of microcrystalline structures of this compound in the resin before the curing process [21].

From the analysis of functional groups, the absorption bands shown by the uncured Aerotuf resin agree with those reported in literature for a mixture composed of epoxide, amine, and MI compounds, which gradually react during the stages of the curing process, as will be discussed later. The epoxy resin modified with imide groups such as MIs have demonstrated to have high cross-linking ability, specific strength and specific modulus, lower water absorption, and superior thermal and flame-retardant properties compared with conventional epoxy resins [21,22,23,24,25]. Such properties are convenient for the fabrication of aeronautical components.

**Figure 2:**
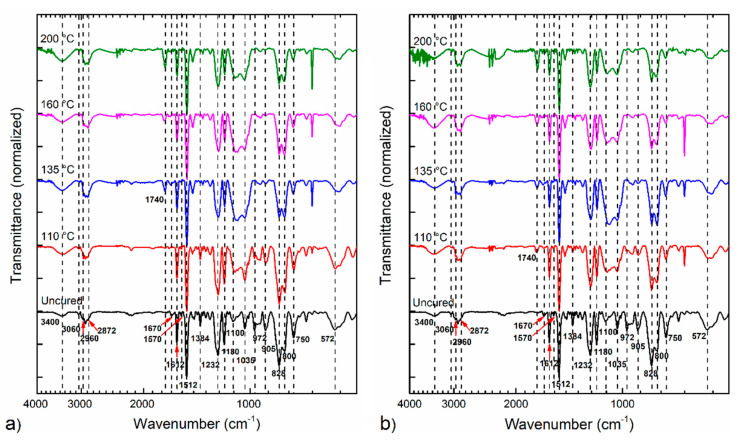
FTIR spectra of Aerotuf resin cured at different temperatures and times: (**a**) 15 min and (**b**) 30 min.

To have a better understanding of the Aerotuf resin curing process and to determine the best processing conditions of the composite laminates, an investigation of its physical behavior for different temperatures (110, 135, 160, and 200 °C) and curing times (15 and 30 min) was carried out. Figure 2 shows the normalized FTIR spectra obtained for the analyzed samples including the spectrum of uncured sample. The transmittance spectra were normalized from 0 to 1, taking the band intensity at 1512 cm^−1^ as a reference (assigned to the C–C stretching of the aromatic ring) as this band does not change along the reaction path [2,16,26,27]. As seen in Figure 2a, samples cured for 15 min show that the O–H absorption band at 3400 cm^−1^ tends to increase as the curing temperature rises from 110 to 200 °C. For a curing temperature of 135 °C, it is observed that the appearance of a band around 1740 cm^−1^ attributed to carbonyl absorption of MI units, which gets more intense as the curing temperature rises. The bands assigned to amine and imide groups at 1670, 1570, and 1384 cm^−1^, and those of epoxide group (972 and 905 cm^−1^), diminish prior to disappearing as the curing temperature rises. Moreover, the band at 828 cm^−1^, associated with the H-C= unsaturation present in MI rings, exhibits a reduction in intensity when the curing temperature is increased. Bands located at 2960, 2872, 1100, 1035, and 800 cm^−1^ show changes in intensity that are attributed to the presence of mold release agent in the analyzed samples; for this reason, the changes observed in such absorption bands are not considered in this study.

Figure 2b shows the FTIR spectra of the samples cured for 30 min. Notice from Figure 2b similar changes in the intensities of the active groups in comparison with the samples cured during 15 min. Furthermore, the effects observed in the cured resin when varying the curing temperature, an increase in the curing time from 15 to 30 min does not produce a clear difference in the intensities of the absorption bands, preventing a comprehensive comparison between both curing times.

### 3.2. Resin Curing Reaction Behavior

**Figure 3:**
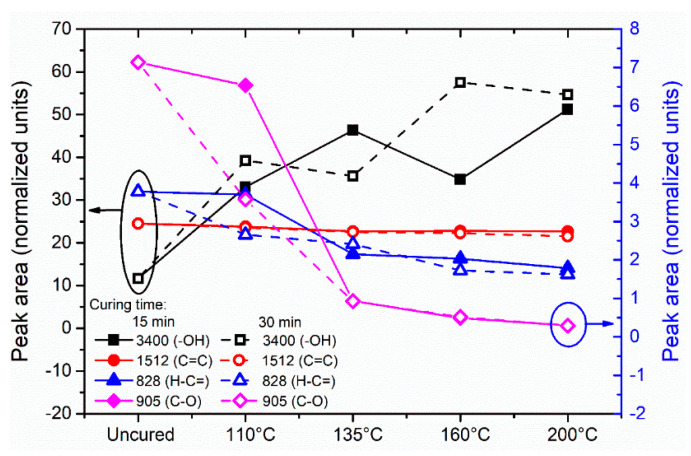
Calculated peak areas of the active functional groups as function of temperature and curing time.

The curing process of epoxy/amine/MI systems involves several chemical reactions that occur either simultaneously or at different stages of the curing process depending on the reactivity of the components and on the processing temperatures [21,28]. The first stage comprises the oxirane ring-opening reaction with active amine groups present in the system. Secondly, etherification reactions of the epoxide group with pendant hydroxyl groups of epoxy resin and hydroxyl groups formed during the reaction could take place. These first two stages are catalyzed by the presence of hydroxyl functionalities created by the amine reactions and/or those added to the formulation as accelerators, which accounts for the autocatalytic nature of the curing process of this resin. Subsequently, MIs are generally cross-linked via a two-step process: (i) Michael addition, i.e., addition reaction of amine groups with double bonds of MI rings; and (ii) radical homopolymerization of MIs either sequentially or simultaneously depending on the curing temperature. To confirm the occurrence of the reactions involved in the curing process of Aerotuf resin, a quantitative analysis is performed to investigate the changes in the area of the IR bands of some active functional group along the different temperatures and curing times. The baseline for each analyzed peak was taken by joining with a straight line the inflection points of the band both ends. For comparison purposes, Figure 3 shows only the calculated peak area for the absorption bands at 3400, 905, and 828 cm^−1^ of the samples cured at different temperatures and times because these are considered to actively participate in the curing reaction. In addition, the area values calculated for the band at 1512 cm^−1^ along the different temperatures and curing times are included to confirm the unchanged behavior of this band. Other active groups such as the bands of amine groups at 1670 and 1570 cm^−1^ are considered unsuitable for quantitative analysis owing to their weak intensity or overlapping with other bands. As seen in Figure 3, the samples cured for 15 min show an increase in the area of the absorption band of hydroxyl groups for increasing temperature values, whereas the area of the bands associated to epoxide groups decreases gradually with increasing temperature values. This tendency is more evident for a curing temperature of 135 °C, which is associated with the coupling of epoxide and amino groups by the oxirane ring opening to produce O–H pendant groups, which act as catalysts for a subsequent etherification reaction between them and the epoxide groups until the complete consumption of the epoxide and amine groups. From the temperature value of 160 °C, the decrease in the peak area values of the epoxy functional group occurs at a lower rate, indicating the almost complete consumption of the species involved in such reactions. When a curing time of 30 min is considered (black dashed line), the increase in the peak area of the hydroxyl functional group reaches a maximum value when a curing temperature of 160 °C is applied, while the area of epoxide peak exhibits a notable reduction that begins at 110 °C. Again, from the use of a curing temperature of 160 °C, the decrease recorded in the epoxide peak area becomes less significant as the temperature increases. Such a tendency confirms that, for a curing temperature of 160 °C, it is possible to achieve an acceptable conversion of the reaction between amine and epoxide groups for adequate curing of the resin.

Another active functional group analyzed in the present work is the absorption band corresponding to the =C–H unsaturation, characteristic in MI rings and located at 828 cm^−1^ in the FTIR spectra of the different studied samples. This absorption band has been used by some authors to monitor the evolution of the coupling of the =C–H unsaturation of MI rings with the amine groups present in the reaction, or the homopolymerization reaction of MI units [21,26]. Both reactions contribute to the complete cross-linking of the resin system. Figure 3 shows the changes in the area values obtained for this band varying the curing temperature and using curing times of 15 and 30 min. As observed in Figure 3, the peak area associated to this functional group shows a monotonic reduction as the curing temperature increases, indicating a gradual consumption of MI double bonds. However, when a curing time of 30 min is considered, the reduction in the peak area of =C–H unsaturation is observed to rise from 110 °C, evidencing the influence of curing time to promote the occurrence of cross-linking reactions of MI compounds. However, it should be noted that, after a curing temperature of 135 °C in both curing times, the reduction in the peak area of this active group is less significant for increasing temperature values because the reaction of MI double bonds is almost completed. The consumption tendency observed for this =C–H unsaturation does not reach minimum values, as in the case of the epoxide groups, which could be because of the overlapping of the absorption band with the assigned non-reactive functional groups corresponding to *p*-substituted aromatic rings located in the same position that hinder the accurate monitoring of the reaction of MI units.

### 3.3. Curing Degree Quantitative Analysis

**Figure 4:**
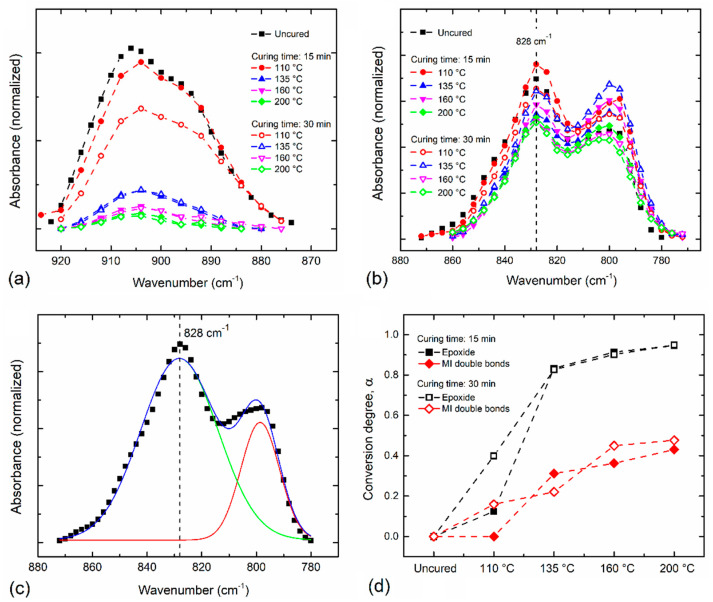
Comparison of FTIR absorbance bands for (**a**) epoxide group at 905 cm^−1^ and (**b**) maleimide (MI) double bonds. (**c**) Deconvolution of FTIR absorbance band into gaussian components performed for area calculation of band at 828 cm^−1^, where the scatter plot corresponds to experimental data. Here, solid lines indicate the computed fitting curves. (**d**) Conversion degree *α* for different curing times calculated for the epoxide group and MI double bonds as a function of the curing temperature.

Based on the chemical changes observed from the absorption bands of the epoxide groups and MI double bonds, for different curing temperatures and times that occur during the reaction mechanism process, it is possible to define the extent of the reaction in terms of epoxide groups and MI double following the Beer–Lambert’s law equations [18,21,27]:

(1) $$\alpha_{E}=1-A_{Et}A_{R0}/\left(A_{Rt}A_{E0}\right),$$

(2) $$\alpha_{MI}=1-A_{MIt}A_{R0}/\left(A_{Rt}A_{MI0}\right),$$

where *α_E_* and *α_MI_* are the conversion degree in terms of epoxide groups and MI double bonds, respectively. *A_Et_*, *A_MIt_*, *A_E_*_0_, and *A_MI_*_0_ refer to the area under the epoxide peak at 905 cm^−1^ and the peak area of =C–H unsaturation of MI at 828 cm^−1^ calculated from the absorption spectrum at time *t* and 0 min, respectively. *A_R_*_0_ and *A_Rt_* correspond to the area under the reference peak at the beginning of the curing process (*t* = 0) and after a certain curing time (*t*); in our calculations, the band at 1512 cm^−1^ was taken as a reference band. Figure 4a shows the comparison of the FTIR absorbance bands of the epoxide group for different temperatures and curing times. As seen in Figure 4a, the intensity of the epoxide band reduces as the reaction temperature increases. In the samples cured during 15 min, a remarkable reduction in the intensity of this band is observed after 135 °C, while in the samples cured during 30 min, the reduction in the band intensity begins after 110 °C. As the temperature increases, the decrements observed in intensity are similar for both curing times. In the case of the band at 828 cm^−1^ (corresponding to the unsaturation of MI units), as shown in Figure 4b, a reduction in its intensity, as the curing temperature increases, is observed; however, it is less significant than that observed for the epoxide group.

The area under the curve for each band is found and used for the estimation of the conversion degree *α* in terms of epoxide groups (*α_E_*), and MI double bonds (*α_MI_*) for different temperatures and curing times, as shown in Figure 4c,d. In both functional groups, the conversion degree gradually increases as the curing temperature rises; however, in terms of epoxide groups, *α* is notably higher compared with the conversion of MI double bonds. After 135 °C, the increase in the curing time seems not to influence the *α_E_* values, showing a conversion almost equal for the samples cured during 15 and 30 min. When the curing temperature rises to 160 °C, it is possible to reach a conversion degree of 0.91, whereas, in the sample cured at 200 °C, a small additional increase reaching 0.95 conversion is obtained. Accurate values for high conversion degree obtained by the integration of the IR band of the epoxide group have been reported in [9,21,29]. These accurate values are mostly due to the well-defined band characteristics when compared with others in the fingerprint region that could influence its behavior during the curing process, and to the small integration error obtained during the calculations. Therefore, the conversion degree obtained from the changes in the band intensity of the epoxide group along the different temperatures and curing times could represent a good approximation of the real conversion of Aerotuf resin.

On the other hand, the value of the conversion rate computed from Equation (2) for the reaction of MI double bonds tends to increase as a function of the temperature and curing time; however, these conversion rate values are about 50% lower than those calculated in terms of the epoxide groups using Equation (1). After a curing temperature value of 160 °C is applied, the increase in the conversion degree of MI is almost negligible, but, in this case, when the curing time is applied during 30 min, a slight increase in the *α_MI_* is observed, reaching a maximum conversion of 0.48. The lowest values of *α*_*MI*_ in comparison with those estimated for the epoxide groups can be attributed to the overlapping of the band at 828 cm^−1^ with the non-reactive absorption of *p*-substituted aromatic rings. In this case, it is difficult to quantify the changes that occur in the intensity of such band for increasing temperatures and curing times, leading to inaccurate calculations of conversion degree of MI double bonds. Moreover, the reaction of MI double bonds possibly provides a small contribution to the overall curing process owing to the presence of small quantities of such a compound in the resin system, which makes it difficult to follow its behavior during the curing process by FTIR analysis. In any case, the use of the absorption band at 828 cm^−1^ results inappropriate to study the Aerotuf curing process.

Based on the tendency observed in the conversion degree determined for both functional groups, and despite the differences in the level of total consumption for both groups for different curing temperatures and times, it is possible to assume the occurrence of two independent reaction pathways during the Aerotuf curing process. One due to the reaction of the epoxide groups with amine groups and pendant hydroxyl groups; and the other due to the coupling of the =C–H unsaturation of MI rings with the amine groups, or the homopolymerization of MI units.

We shall next investigate the mechanical properties of composite laminates produced for different curing temperatures and times.

### 3.4. Influence of the Curing Temperature and Time on the Mechanical Properties of [±45°] WCFF/Aerotuf Composite Laminates

**Figure 5:**
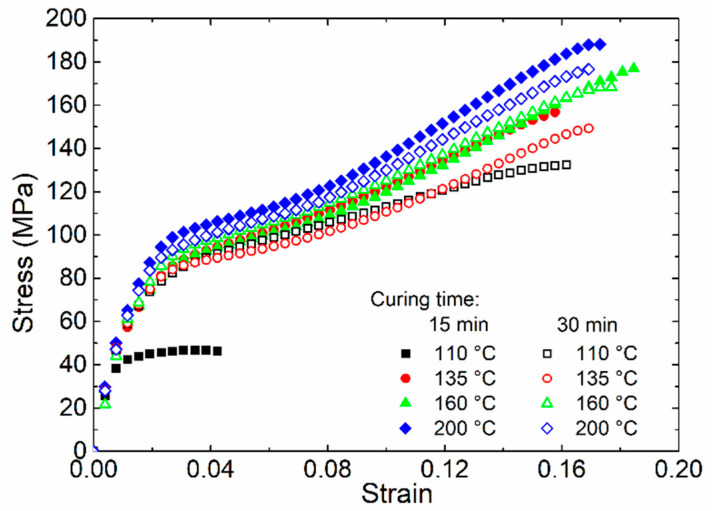
Stress–strain curves for the [±45°] WCFF/Aerotuf composite laminates cured at different temperature and times.

The mechanical behavior of [±45°] WCFF/Aerotuf composite laminates prepared at a fixed pressure of 8.3 MPa and using different curing temperatures and time was evaluated performing samples tensile tests. Figure 5 shows the stress–strain curves of the tested composite samples that exhibit the elastic and plastic regions, characteristic of polymer materials, except for that corresponding to the composite laminate cured at 110 °C during 15 min, which exhibits a premature failure at low strain, attributed to the insufficient curing degree (~0.12), as previously discussed. The general trend observed in most of the tested samples indicates that, in the elastic region, the load is supported by the resin and the carbon fibers; as the samples elongation increases to reach the plastic region, the resin is the main responsible of the load transfer until the failure occurs by plastic deformation. Such a trend is typically observed in angled WCFF/epoxy composites owing to their pseudo-ductile behavior [30]. For the samples produced considering both curing times of 15 and 30 min, the maximum strain of the composite samples increases as the curing temperature rises up to 160 °C. These results are consistent with the quantitative analysis of curing behavior obtained by FTIR analysis, confirming that, at such a temperature, it is possible to achieve an adequate conversion degree (>0.9) that allows to achieve improved mechanical performance for Aerotuf resin.

The measured values of the ultimate tensile strength (UTS) and elastic modulus, *E*, for [±45°] WCFF/Aerotuf composite laminates are summarized in Table 2. As observed, the UTS increases monotonically as a function of the curing temperature regardless of the curing time (15 or 30 min), yielding a maximum UTS value of 180.7 MPa. Notice that the composite laminate sample cured at 110 °C exhibits a remarkable increment in the UTS value when a curing time of 30 min is considered. It is also seen in Table 2 that, for increasing curing temperatures, the application of higher curing time does not produce significant differences in the UTS values in comparison with those obtained when the composite laminate samples are cured during 15 min. In general, the coefficient of variation (CV) values of the UTS for both curing times are relatively low (<10%) and do not display substantial changes from increasing curing time. From the asymptotical tendency described by the UTS values of the composites samples cured during 15 min, one must conclude that curing temperatures higher than 160 °C produce a marginal increment in the UTS value close to 5% when compared with the UTS values obtained from the composite laminates cured at 160 °C. Such a tendency agrees with the values of conversion degree of the samples found by FTIR analysis, evidencing that applying a curing temperature of 160 °C during 15 min could be enough to achieve a good mechanical performance of the composite laminates. The elastic modulus values experimentally measured from the composite laminate samples are listed in Table 2. One can see from Table 2 that the elastic modulus values are almost independent of the curing temperature and time (within the experimental error). The CV values of the elastic modulus are found to be between 1.3 and 7.6%, being relatively similar for both curing times. Apparently, the variation in the processing conditions does not produce significant changes in the recorded elastic modulus values of the composite laminates; however, by considering the standard deviation values of these composite samples, such a tendency cannot be completely confirmed; therefore, a more extensive analysis about this property is needed. However, the experimental values of the UTS and elastic modulus of the produced composite samples are similar to other [±45°] WCFF/epoxy composite laminates developed for structural applications using different curing resin [30,31,32].

Thus, the mechanical performance of the produced composite laminate samples depends on great extent of the resin curing/cross-linking degree, as confirmed in [3,4,33,34,35]. In fact, as the curing degree increases, it is expected to have better interfacial strength between carbon fibers and resin and, therefore, an efficient stress transfer through the material.

## 4. Conclusions

The curing process of an epoxy-based resin—Aerotuf 275-34^TM^—cured at different temperatures (110, 135, 160, and 200 °C) and times (15 and 30 min) was investigated using the FTIR technique to identify the processing conditions of [±45°] WCFF/Aerotuf composite laminates produced by compression molding. Then, tensile test were performed on the produced samples to correlate their mechanical properties with the epoxy resin curing degree. In summary, the main article findings are as follows:

- The reaction mechanism that occurs in Aerotuf resin curing process consists of two independent reactions: (a) the consumption of the epoxide groups and (b) the reaction of the =C–H unsaturation of MI units either with the amine groups or by homopolymerization, which are influenced by the reaction temperature and the curing time.
- The baseline band absorption located at 905 cm^−1^ can be used determine the resin curing/cross-linking degree in terms of epoxide groups.
- An epoxy resin curing temperature of 160 °C during 15 min is enough to achieve an adequate conversion degree of 0.91 in terms of epoxide groups representing a good approximation of the real conversion of Aerotuf.
- Tensile tests confirmed that the ultimate tensile strength values of the produced [±45°] WCFF/Aerotuf composite laminates samples achieved the best mechanical performance when using an epoxy resin curing temperature of 160 °C during 15 min.

This article elucidates how the curing/cross-linking degree of Aerotuf resin influences the mechanical performance of composite laminates; therefore, these results can be used to improve the mechanical properties of composite laminates with different carbon fiber orientation angles.

## Figures and Tables

**Table 1:** Band assignments of Fourier-transform infrared spectroscopy (FTIR) spectrum for uncured Aerotuf resin.

Wavenumber (cm^−1^)	Band Assignment
3400	O–H stretching and symmetric stretching of primary amine
3060	C–H stretching of epoxide group
2960, 2920, 2872	C–H stretching of CH_2_ and CH_3_ aromatic and aliphatic
1670	N–H bending of primary amine
1612	C=C stretching of aromatic ring, N–H bending of primary amine
1570	N–H bending of primary amine
1512	C–C stretching of aromatic ring
1384	C–N stretching of imide
1232	C–O–C stretching of ether linkage
1180	C–O stretching of aromatic ring
1100	Aromatic stretching
1035	C–O–C stretching of ether linkage
972	Glycidyl ether of oxirane ring
905	C–O stretching of oxirane ring
828–800	H–C= out-of-plane bending of MI ring and 1–4 substituted aromatic ring
750–572	C–H out-of-plane of aromatic ring

**Table 2:** Measured values of the ultimate tensile strength (UTS) and elastic modulus, *E*, for [±45°] woven carbon fiber fabric (WCFF)/Aerotuf composite laminates manufactured at different curing temperatures and times.

Curing Time (min)	Curing Temperature (°C)	UTS (MPa)			*E* (GPa)		
		Average	SD	CV (%)	Average	SD	CV (%)
15	110	45.1	1.5	3.3	6.4	0.2	2.5
	135	149.7	7.4	4.9	6.1	0.2	3.6
	160	172.0	5.0	2.9	6.4	0.2	3.7
	200	180.7	8.7	4.8	6.2	0.3	4.7
30	110 135 160 200	131.3 141.3 153.7 174.3	1.2 6.7 13.3 4.6	2.6 4.4 7.7 2.6	6.4 6.1 6.0 5.9	0.1 0.4 0.2 0.4	1.3 6.3 3.6 7.6

SD: standard deviation. CV: coefficient of variation. UTS: ultimate tensile strength.

## Data Availability

Not applicable.
